# When can Muslims withdraw or withhold life support? A narrative review of Islamic juridical rulings

**DOI:** 10.1080/11287462.2020.1736243

**Published:** 2020-03-22

**Authors:** Afshan Mohiuddin, Mehrunisha Suleman, Shoaib Rasheed, Aasim I. Padela

**Affiliations:** a Initiative on Islam and Medicine, Program on Medicine and Religion, The University of Chicago, Chicago, IL, USA; b Department of Internal Medicine, University Hospitals Regional Medical Center, Macomb Township, MI, USA; c Centre of Islamic Studies, University of Cambridge, Cambridge, UK; d Department of Internal Medicine, Henry Ford Macomb Hospital, Chicago, IL, USA; e Section of Emergency Medicine, Department of Medicine, The University of Chicago, Chicago, IL, USA; f MacLean Center for Clinical Medical Ethics, The University of Chicago, Chicago, IL, USA

**Keywords:** Withholding, withdrawal, Fatwa, end of life care, Islam Muslim

## Abstract

When it is ethically justifiable to stop medical treatment? For many Muslim patients, families, and clinicians this ethical question remains a challenging one as Islamic ethico-legal guidance on such matters remains scattered and difficult to interpret.

In light of this gap, we conducted a systematic literature review to aggregate rulings from Islamic jurists and juridical councils on whether, and when, it is permitted to withdraw and/or withhold life-sustaining care. A total of 16 *fatwās* were found, 8 of which were single-author rulings, and 8 represented the collective view of a juridical council. The *fatwās* are similar in that nearly all judge that Islamic law, provided certain conditions are met, permits abstaining from life-sustaining treatment. Notably, the justifying conditions appear to rely on physician assessment of the clinical prognosis. The *fatwās* differ when it comes to what conditions justify withdrawing or withholding life- sustaining care. Our analyses suggest that while notions of futility greatly impact the bioethical discourse regarding with holding and/or withdrawal of treatment, the conceptualization of futility lacks nuance. Therefore, clinicians, Islamic jurists, and bioethicists need to come together in order to unify a conception of medical futility and relate it to the ethics of withholding and/or withdrawal of treatment.

## Introduction

Every culture has its own understanding of the events near the end-of-life, and the appropriate rites and rituals to perform at that time. For Muslims, beliefs, practices and rites nearing the end-of-life centre on this phase being a transition period that precedes an everlasting afterlife (Sheikh, 1998). These understandings influence the significance attached to the liminal state between life and death, and the ethical duties and obligations that ensue. In this way, Muslim patients, families, and physicians carry their beliefs and values into the clinical encounter and may seek to negotiate healthcare pathways that align with their religious values, and at the same time, cohere with the conventions, professional standards, and expectations of clinical care. Thus when negotiating clinical care goals at the end-of-life, and when making decisions about withholding and withdrawing of life-sustaining care, clinicians, patients and families may look to the ethical guidelines sourced within their faith tradition (Padela & Mohiuddin, 2015).

For Muslims, religious beliefs and values are informed by the *Sharī’ah*, which etymologically means “way to the water” and refers to Islamic moral law. The *Sharī’ah* provides Muslims with scripturally-sourced guidance about obligations and duties in all facets of life, including healthcare at the end-of-life. Islamic theologians and jurists through the ages have developed rules of the *Sharī’ah*, and the science that is at the centre of this endeavour is moral theology^1^ (*uṣūl al-fiqh*). This science identifies the sources of ethico-legal knowledge, and lays down discursive rules for ethico-moral reasoning. The end-product of Islamic ethical deliberation through the application of *uṣūl al-fiqh* is *fiqh* (law).

*Uṣūl al-fiqh* is based on material and formal sources. The material sources are the Quran (considered to be the literal word of God communicated to the Prophet Muhammad) and Sunnah (the practices, sayings, and tacit approvals of the Prophet Muhammad). Trained jurists interpret these two primary sources and derive contextualised rulings for the Muslim community. The formal sources include *ijmā’* (consensus-based agreement) and *qiyās* (analogical reasoning). These four sources, although sometimes differently prioritized and applied, are considered primary to generating moral law by the four extant Sunni schools of Islamic law. Importantly, multiple secondary tools also help inform ethico-legal assessment.^2^

In this paper we will be focusing on two particular outputs from Islamic ethico-legal reasoning; the *fatwā*, a nonbinding ethico-legal Islamic opinion by a qualified Islamic jurist consult, and *qarār*, an Islamic opinion issued by a committee of Islamic jurists.^3^ Traditionally, ethico-legal deliberations in Muslim communities, both scholarly and lay, centre around juridical rulings of past and present jurists. Consequently academics have emphasised the use of *fatwā* literature as a source of information within the developing field of Islamic bioethics.^4^ Rispler-Chaim aptly describes *fatwā* as a “dialogue between lay people and muftis,” (Rispler-Chaim, 1993) where religious teachings are constructed by scholars and mediated towards the public. *Fatwās* and scholarly engagement is a critical tool for stimulating a broader ethical discourse that begin with questions of religio-legal endorsement. Thus, as Brockopp and Eich explain, “scholars (may) represent (the) Islamic law not as static, immutable entity but rather as a discursive tradition that seeks to apply God's unchanging law to the ever changing world we live in” (Brockopp & Eich, 2008).

Unless backed by a state authority, *fatwās* are non-binding. Thus, individuals are free to choose amongst these ethico-legal opinions. Moreover the plurality of Islamic moral theology results in there being a plethora of *fatwās* addressing similar questions. To some, the numerous *fatwās* may appear as an unsystematic and bewildering variation in views, however this characteristic is lauded by many as an expression of the ethico-legal plurality of Islam. Truly, beyond the small range of unambiguous or prescribed duties, scholars are called upon to navigate the large spectrum of emerging concerns or to revisit previously encountered problems using a fresh approach. *Fatwās* therefore offer a rich research source from which Islamic ethico-legal reasoning and scholarly attempts to approach modern problems can be understood.

With the recent advancement of the biomedical sciences, and the resulting proliferation of ethical challenges encountered by patients and health professionals, Islamic jurists (known as *muftis* or *faqīhs*) are increasingly approached to provide religious guidance on complex decisions. This is particularly true in the arena of end-of-life care where Muslim physicians, families, and patients struggle with decision-making. They struggle in finding actionable guidance, in part, because there are few papers that analyse the various juridical positions on end-of-life care ethics and present them in an accessible fashion (Padela & Mohiuddin, 2015). In particular, there has been no summative review that we are aware of that covers the various Islamic bioethical views on withholding and withdrawal of interventions near the end-of-life. Neither has there been an attempt to identify the key concepts upon which the juridical opinions hinge, and the conditions upon which withholding and/or withdrawal of life support is justified. This paper fills in this gap by providing a narrative review of *fatwās* on the conditions which justify withdrawing and/or withholding of life-sustaining treatments.

## Methods

### Search strategy

**Figure 1.**
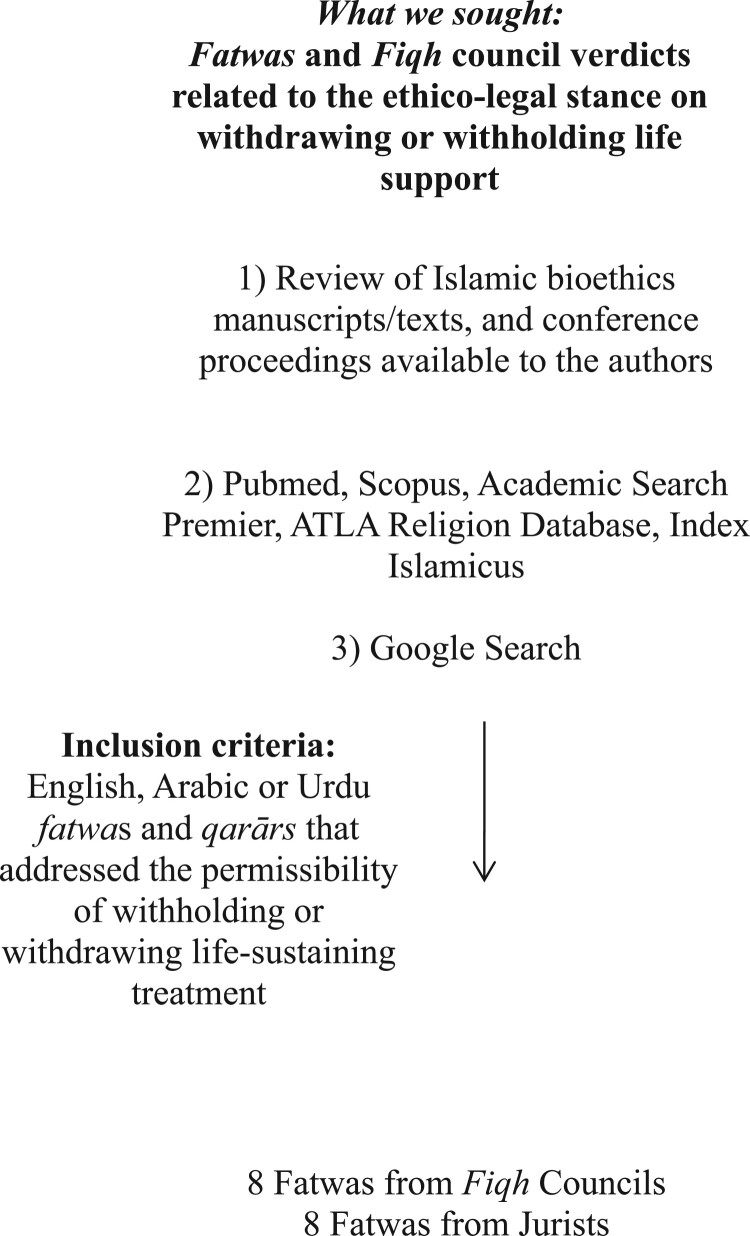
Search strategy for Sunni fatwās of muftīs/faqīhs and Muslim fiqh councils

Our literature search involved three stages. The first stage of the search was the review of Islamic bioethics manuscripts/texts and conference proceedings that were available to the authors to uncover cited juridical rulings on the topic. The second stage involved Google searches for *fatwā* online (performed on 09/19/2014). The opinions of physicians or other scholars (non-jurists) were not considered to be *fatwās*. Juridical rulings related to end-of-life care within these sources were identified and obtained. The third stage of the search used Pubmed, Scopus, ATLA Religion Database, and Index Islamicus databases. These databases were chosen in order to have a comprehensive search spanning the fields of medicine, social sciences and religion. The search string for the literature databases included the following terms: “passive euthanasia” or “life support care” or “life support” or “life sustaining treatment” or “critical care” or “withholding treatment” or “withdrawing treatment” or “withholding care” or “withdrawing care” or “end of life” or “terminal care” or “ethics” as mesh terms/main subject headings or text words in all fields. These were linked to “Islam(ic)” or “Muslim(s)” as main subject headings (exploded) or text words in all fields. Articles in the English language that expressed views on the issue of withholding and/or withdrawing medical treatment or life support were included. Importantly, *fatwās* were included only if they were ascribed to trained *muftīs* or *faqīhs*, or to national and international Muslim *fiqh* councils. Figure 1 illustrates the search strategy. Each *fatwā* was analysed for the exact medical intervention in question, whether or not they made a distinction between withholding and withdrawing, and any justifying conditions that were cited.

## Results

Our search yielded 8 *qarārs* and 8 *fatwās*. A summary of the juristic opinions regarding withholding and withdrawal of treatment are presented below. Additional topics covered within the juridical analyses included comments about brain death, pain relief, living wills and resource allocation. Prior to delving into our results we would like to provide brief background details on the jurists and councils whose opinions were found. Of note, two of the individual jurists have past or current leadership positions on the *fiqh* councils with published *qarārs*. Shaykh Qaradawi currently chairs the European Council of *Fatwā* & Research (European Council for Fatwa, and Research, 2000). Ebrahim Desai is the former head of Dar al-Ifta of Madrasah In’aamiyyah, and the current head of the *Fatwā* Department of Jamiatul Ulama Kwa Zulu Natal.^5^

### Islamic Fiqh Academy of the Organisation of the Islamic Conference (IFA-OIC)

The IFA-OIC consists of Muslim jurists appointed by governments to represent their countries (43 of 57 OIC countries are represented). This core group is supplemented by other prominent jurists and/or topical experts recommended by current members (Padela et al., 2011).

### Islamic Organisation of Medical Sciences (IOMS)

Founded in the 1990s IOMS

was formed to fill a need for the Muslim ‘Ummah’ (worldwide nation), to clarify the Islamic point of view of certain medical practices, to collect Islamic medical heritage and to determine how to apply it to modern day medical practice. (Rahman, 2000)

The organisation focused on “including the spiritual component in the definition of the human being at World Health Organization (WHO)” (Rahman, 2000). IOMS has published a series of medical codes of ethics from the Islamic ethico-legal perspective.^6^ This group routinely gathered Islamic jurists from around the world to opine on medical ethics issues at their *fiqh* conferences.

### Islamic Fiqh Council of the Muslim World League (IFC-MWL)

The Islamic *Fiqh* Council was founded in 1977 as an independent entity within the MWL, an international non-governmental Islamic organisation based in Saudi Arabia. It comprises of a select group of Islamic jurisconsults who are committed to elucidating the rulings of the *Shari’ah* regarding problems and calamities faced by Muslims.^7^

### Islamic Fiqh Academy India (IFA-I)

Founded in 1989, the IFA-I's primary objective is

to find solutions for the contemporary problems brought up by the developments and changes in social, political, economic, industrial and technological spheres of life, in the light of the guidelines provided by the Quran and Sunnah and deliberations and interpretations of the companions of the Prophet and other pious classical jurists and scholars.^8^

Although based in India, its scope extends internationally, and the organisation seeks to co-ordinate efforts with other juridical bodies.

### Saudi Permanent Committee for Islamic Research and Fatwās

The Saudi Permanent Committee for Islamic Research and *Fatwā*s is a committee established by royal decree in 1971 by King Faisal ibn Abd al-Aziz of Saudi Arabia. It issues *fatwās* relevant to all aspects of life (regarding creed, worship, and social issues) both in print and online. Its members are drawn from the most senior Sunni scholars of *fiqh* in Saudi Arabia. Its head is the Grand Mufti of Saudi Arabia.^9^

### Madrasah In’aamiyyah

The Department of Jurisprudence at Madrasa In’aamiyyah, an institute of Islamic education based in South Africa, is responsible for “assisting people with regards to their day to day queries regarding issues of Shariah.”^10^

### European Council for Fatwā & Research (ECFR)

ECFR was established in 1997. It is constituted of scholars across Europe and is led by Shaykh Yusuf al-Qaradawi. It is headquartered in Dublin with branches in the Middle East. The council's aims is to supply interpretations of Islamic law tailored to Muslim minority communities.^11^

### Darul Uloom Zakariyya

Darul Uloom Zakariyya is a school established in 1983 near Johannesburg, South Africa, which aims “to provide and impart higher Islamic Education to the Muslim community.”^12^

### Jamiatul Ulama Kwa Zulu Natal (KZN)

Jamiatul Ulama KZN is a body of Islamic scholars established in 1955 in South Africa. Its primary aim is the “preservation, protection and propagation of true and pristine Islam official body of religious, educational and other affairs of the Muslim Community of KwaZulu-Natal.”^13^ Its head of the *Fatwā* Department is Mufti Ebrahim Desai.

## Individual *muftīs*/*faqīhs*

Hani al-Jubayr is an Islamic scholar based in Saudi Arabia and works for the Jeddah Supreme Court.^14^

Jad al Haqq Ali al Haqq (1917–1996) was the former Grand Mufti of Egypt, former Minister of Religious Endowments, and former Grand Shaykh of al-Azhar.

Yusuf al-Qaradawi was born in Egypt in 1926 and is primarily based in Qatar. He is an al-Azhar educated jurist, affiliated with IslamOnline, and chairman of the International Union of Muslim Scholars.^15^ As of 2018, he is also the chair of ECFR.

Ali Gomaa was born in Egypt in 1952 and served as the eighteenth Grand Mufti of Egypt (2003–2013), a position of religious authority that is second only to the sheikh of Al-Azhar University.^16^ He is a prominent scholar, jurist, and public figure based in Egypt.

Muhammad Salih al-Munajjid was born in Syria in 1960, and lives in Saudi Arabia. He runs IslamQA, a website that publishes his *fatwā*s. IslamQA.info is available in 16 languages including English, Arabic, Bangla, Chinese, Russian, French, and Spanish.^17^

Ebrahim Desai is a South African Islamic schoar born in 1963. He is the founder of the *fatwā* website, “AskImam,” the former head of Dar al-Ifta of Madrasah In’aamiyyah, and the current head of the *Fatwā* Department of Jamiatul Ulama Kwa Zulu Natal and the Darul Iftaa Mahmudiyyah in Durban, South Africa.^18^

Muhammad ibn Adam al-Kawthari was born in Leicester UK in 1976. He is a teacher of various traditional Islamic sciences in Leicester, London and other locations, and director and researcher at the Institute of Islamic Jurisprudence (Darul Iftaa), which “aims to provide insight into the Islamic perspective on personal, social, and global issues.”^19^

Ikram ul Haq is the imam of Masjid Al-Islam in Rhode Island, USA and founder and director of *Fatwā* Center of America (askamufti.com), which seeks to “cater the needs of global Muslim community in answering their questions pertaining to religious life and spirituality.”^20^ The questions are primarily answered by Ikram ul Haq.

### Juridical opinions on the withdrawing and withholding of treatment at the end-of-life

All the *fatwās* except Shaykh Hani al-Jubayr's permit the foregoing of medical care provided certain conditions are met. Discontinuation of care was judged permissible in situations where death of the patient seemed inevitable. The *fatwās* expressed this situation with terms such as “futile or useless” (IOMS), “when sickness gets out of hand, and recovery happens to be tied to a miracle,” (Qaradawi),^21^ “physicians have lost hope for life,” (IFA-India, 2014) “no longer hoped that such a person will be cured,” (Gomaa)^22^ or “no chance of survival” (Desai).^23^

Shaykh Hani al-Jubayr, on the other hand, does not find Islamic law to permit the foregoing of medical care. It is important to note the specific circumstances within which he makes his ruling, and that the context differs from that of the other scholars. In response to a question of whether or not a patient who is terminally ill and suffering from great pain can request to have his life ended, Shaykh Hani Al-Jubayr responds saying that it is impermissible. He states that a physician will be sinning if he assists that patient in his death. He continues to say

there is no difference in this regard whether the death of the patient is brought about by turning off his life support, or withholding medications that the patient needs to stay alive, or by administering medications that induced death. All such procedures are unlawful. They may not be carried out by anyone, nor may anyone give his consent to them. (al-Jubayr, 2012)

Shaykh Hani Al-Jubayr's view contrasts with others’ opinions. For example, Shaykh Yusuf Qaradawi not only permits foregoing of medical care but actually recommends it at times, including situations of terminal pain (see section below).^24^

Some *fatwās* distinguish between two categories of end-of-life treatment: life support and ancillary interventions. The former primarily refers to treatments such as assisted mechanical ventilation. The latter includes nutrition, hydration, pain control, antibiotics, and such therapeutics. *Fatwās* that make this distinction rule that while life support treatment is permissible to stop, ancillary treatment should be continued. For example, Shaykh Ali al-Gomaa ambiguously writes that while medical equipment can be removed when hope for recovery no longer exists, it is not permissible to remove equipment that has “another purpose, like the removal of fluid to improve respiratory health.”

In general, although futility considerations appear to allow for withdrawal and withholding of life-sustaining treatment, the *fatwā*s avoid providing specific examples of indications in which treatment would be considered futile. This hesitance appears purposeful, giving considerable flexibility to clinicians in their decision making.

In this context, the issue of familial decision-making authority was addressed by the *fatwās* of the Saudi Permanent Committee. These responded to queries about the extent to which a family's request for resuscitation be honoured, when doctors deem such resuscitation to be futile. The Committee ruled that as long as three specialists agree that resuscitation is futile, opposition from the family members may be overruled.^25^ Indeed this notion of clinician consensus over futile care is reflected in laws that permit unilateral decisions about DNR (Do Not Resuscitate) status as well as withdrawal and withholding of life support. Another jurist, Mufti al-Kawthari concedes that it is permissible to withdraw treatment in futile situations with the consent of the patient or his immediate relatives, but does not comment on whether such consent is necessary or merely recommended.

Two jurists discussed withdrawing and withholding from the perspective of classical stances on the moral status of seeking medical treatment. Both Shaykh al-Qaradawi and Mufti al-Kawthari identify four opinions that classical Islamic authorities have advanced on the moral status of seeking clinical care: obligatory, recommended, permissible, and that it is more virtuous to abstain from seeking treatment.^26^^,^ ^27^ They both note that the majority of jurists hold the opinion that seeking treatment is simply permissible, with no sin on a person that chooses not to seek treatment. Hence, they argue that the decision for a patient to withdraw care is permissible since it was never more than merely permissible to seek care to begin with.

With respect to conceptual, and ethico-legal, distinctions between withdrawal and withholding of treatment, there appears to be little deliberate distinction made. Only Madrasah Inaamiyyah differentiated between the two actions explicitly, allowing for the withholding of medical treatment, but leaving open the decision on whether the withdrawing of the respirator was ethico-legally permitted.^28^ Broadly, the justifying conditions were found to be similar including: useless/futile care, terminal/inevitable death and depressed neurological function. The only exception was that intractable pain was cited as an acceptable justification for the withdrawal of treatment and not for the withholding of treatment.^29^

The individual and council opinions on the withholding and withdrawing of treatment at the end-of-life are summarised in Tables 1–4.

**Table 1.** Decisions of organisations on withholding and withdrawal of medical care.

Year	Organisation		Form of decision	When can care be withheld?	When can care be withdrawn?	Type of medical care in question
1977	Council of the Islamic Jurisprudence Academy of Muslim World League	International, based in Saudi Arabia	*Qarār*	–	Brain Death	Life-support equipment
1981	IOMS (Islamic Organization of Medical Sciences) Islamic Conference	International, represents diverse Islamic schools of law and theology	Code of Ethics	Recommended if useless; allows treatment abatement for PVS (persistent vegetative state) too	If futile/useless, brain dead (1985); allows treatment abatement for PVS too	Medical treatment
1986	OIC-IFA (Organization of Islamic Conferences Islamic *Fiqh* Academy)	International, represents diverse Islamic schools of law and theology	*Qarār*	–	Inevitable death or brain death	
1989	Saudi Committee for *Fatwā* & Research	Juridical council based in Saudi Arabia	*Fatwā*	If 3 physicians can attest to either: patient unfit for resuscitation, illness unresponsive to treatment and death certain, incapacitated pt or state of mental inactivity, untreatable brain damage, resuscitation would be inappropriate/ineffective	–	Resuscitation (“R” in DNR)
1999	Jamiatul Ulama	Council of Islamic scholars based in South-Africa	*Fatwā*	If no chance of survival*	–	Life-support
2010	Islamic *Fiqh* Academy, India	New Delhi	*Qarār*		When physicians are hopeful that the patient's natural respiratory system will be restored, but the patient or relatives do not have the assets to afford continuation If physicians have “lost hope for his life,” the relatives may ask for the removal	Artificial respirator system
	ECFR (European Council for *Fatwā* & Research)	Led by Shaykh Yusuf Qaradawi	*Fatwā*	–	Loss of *idrīk* (that part of the brain that perceives)	Artificial resuscitation apparatus
	Darul Uloom Zakariyya	Religious school in South-Africa with a *fatwā* department	*Fatwā*	–	Patient cannot stay alive without it (ventilator)	Respirator
	Madrasah Inaamiyyah	Religious school in South-Africa with a *fatwā* department headed by Mufti Ebrahim Desai	*Fatwā*	No chance of survival	Unclear. “Ruling of withdrawal of respirator depends on how the respirator is regarded (an act or an omission).”	Medical treatment

**Table 2.** Decisions of Jurists on withholding and withdrawal of medical care.

	Jurist	Year of birth, Country of origin/work		Can care be withheld?	Can care be withdrawn?	Care in question
	Hani al-Jubayr	Saudi Arabia	Islamic scholar for Jeddah Supreme Court.		No	
2004	Muhammad bin Adam al-Kawthari	UK, 1976	Darul Iftaa, Leicester UK		If futile, no hope for survival/cure	Medical treatment
	Jad al Haqq Ali al Haqq	Egypt (1917–1996)	Former Grand Mufti of Egypt, Minister of Religious Endowments, Grand Sheikh of al-Azhar.	–	If terminally ill	Life-support
	Ebrahim Desai	South Africa, 1963	Runs the *fatwā* website “Ask Imam”, and is the head of Dar al-Ifta of Madrasah In’aamiyyah	–	If terminal*	Life-support
	Ali Gomaa	Egypt, 1953	Former Grand Mufti of Egypt	–	If useless*	Medical equipment utilised to keep a person alive
	Yusuf al-Qaradawi	Egypt/Qatar, 1926	al-Azhar educated jurist based in Qatar, affiliated with IslamOnline, chairman of the International Union of Muslim Scholars	Recommended if useless	If hopeless recovery, too sick, ever-increasing pain, or brain death	Medical treatment
	Muhammad Salih al-Munajjid	Born in Syria in 1960, lives in Saudi Arabia	Runs IslamQA *fatwā* website	in cases DNR is permitted by Saudi fatwā		Resuscitation (“R” in DNR)
	Ikram ul Haq	USA	Founder and director of *Fatwā* Center of America (askamufti.com), Imam of Masjid Al-Islam in Rhode Island, USA	–	If it worsens or does not improve health	Life-support

**Table 3.** Conditions justifying the withdrawal of medical care.

Conditions justifying the withdrawal of medical care		Category of futility
Useless/futile care	IOMS, Muhammad bin Adam al-Kawthari	
Terminal/Inevitable Death	OIC-IFA, Shaykh Jad al Haqq Ali al Haqq, Mufti Ebrahim Desai, Darul Uloom Zakariyya (respirator)	Imminent Demise
Brain Death	IFC-MWL, OIC-IFA, IOMS, Yusuf al-Qaradawi, Mufti Ebrahim Desai	
Sickness gets out of hand, recovery happens to be tied to a miracle, and ever-increasing pain	Yusuf al-Qaradawi	Qualitative
Loss of *idrāk* (Brain's ability to perceive)	ECFR	
Worsens health	Ikram ul Haq	Physiologic or Qualitative
Lack of personal or relative assets to support continuation	IFA-I	
No hope for cure	Ali Gomaa	Imminent demise

**Table 4.** Conditions justifying the withholding of medical care.

Conditions justifying the withholding of medical care		Category of futility
“Useless” care	IOMS, Yusuf al-Qaradawi	
Terminally ill/ No chance of survival	Madrasah In’aamiyyah, Jamiatul Ulama	Imminent Demise/Lethal Condition
Patient unfit for resuscitation, illness unresponsive to treatment and death certain, incapacitated patient or state of mental inactivity, untreatable brain damage, resuscitation would be inappropriate/ineffective	Saudi Committee for *Fatwā* & Research (resuscitation)	Imminent Demise/Lethal Condition

Several other topics of relevance to end-of-life care ethics were touched upon by these extant juridical decrees. In what follows we comment on these emergent topics.

### Brain death

The topic of brain death was widely discussed among the *fatwā*s. Four *fatwā*s ruled that brain death is an indication for the permissibility of discontinuing life support. While they differ in how they view brain death as death, both Islamic Organization for Medical Sciences (IOMS) and OIC-IFA allow for the removal of life support in the condition of brain death (Ebrahim, 1998). IFC-MWL agrees, and along with OIC-IFA, stipulate that the patient's cardiopulmonary functions must be allowed to cease before pronouncing death, and that three expert physicians must confirm brain death (Ebrahim, 1998, p. 193). Yusuf al-Qaradawi equates brain death with death and cites it as a condition for withdrawal of life-sustaining treatment.^30^

Three *fatwā*s do not explicitly cite brain death, but cite depressed neurological function as a justifying condition for foregoing care. Saudi Committee for *Fatwā* & Research cite incapacitance, state of mental inactivity, or untreatable brain damage as justifying conditions for a Do Not Resuscitate (DNR) order. The European Council for *Fatwā* and Research (ECFR), which is led by Yusuf al-Qaradawi, states that a “loss of *idrāk* (that part of the brain that perceives)” implies that the patient is “dead” or “practically dead” and thus a candidate for withdrawal of life-sustaining treatment.^31^ Mufti Desai refers to ECFR's position in his own *fatwā*.^32^

### Pain control in palliative care/end-of-life

As for the issue of pain control, there were two scenarios that were commented on:

- Severe pain in a patient whose life is not terminal, though the experience of the pain is likely to affect quality of life
- A patient who is in severe pain during the final stage of an illness such that ongoing treatment may in fact be exacerbating their pain, at the expense of maintaining life

In the case of the former, the *fatwā*s that addressed such a situation were unanimous in declaring that the patient may not bring about the end of his or her life for fear of living in pain. Rather doing so would be considered suicide, and any physician assistance in such an endeavour is considered murder.^33^ They advise that patients should endure by exercising patience. Al-Kawthari cites a *ḥadīth* in support of this view in which God forbids entry to Paradise for a man who bled himself to death in order to escape the anguish of a wound that he was suffering from.^34^ In the case of the second scenario, al-Qaradawi identifies pain in such a scenario as an acceptable indication for withholding or discontinuing care.^35^

In the latter case, when sickness accelerates, and recovery happens to be tied to miracle, in addition to ever-increasing pain, most do not say treatment then is obligatory or even recommended (exception Shaykh Hani al-Jubayr – see above). Thus, the physician's act of stopping medication, which happens to be of no use, in this case may be justified, as it helps in mitigating some negative effects of medications, although it may hasten death. This situation is viewed as different from the controversial “mercy killing” (by which the jurists mean assisted dying) as it does not imply a positive action on the part of the physician; rather, it is leaving what is not obligatory or recommended, and thus entails no responsibility.

### What is the Islamic legists’ understanding of scarce resources in the context of withholding and withdrawal of treatment?

IFA-I's ruling was the only *fatwā* reviewed that identified lack of resources as an indication to discontinue care (Islamic Fiqh Academy, India, 2014). It ruled that the relatives of a patient may ask for removal of respiratory support if patients and relatives no longer have the financial resources to continue care, even if physicians may be hopeful that the patient will improve. It is unclear whether the Academy viewed this situation as ideal or a last resort, and what its views were regarding the responsibility of society to provide aid to the patient so that care may continue.

## Discussion

Negotiating clinical care goals at the end-of-life, and making collaborative decisions about the withholding and/or withdrawal of life sustaining treatment, may require taking into consideration the faith commitments of patients, families and health care staff (Bone et al., 1990). Few studies have analysed religious resources commenting on the ethics of such decisions from an Islamic perspective. Here we have provided a review of extant, English-language, *fatwās* relevant to the withholding and/or withdrawal of life support to attend to the questions of whether, and when, life-sustaining treatment may be justifiably withheld or withdrawn. With respect to the first question, all save one of the *fatwās* evaluated here deem it Islamically permissible to withdraw or withhold life support provided several conditions are met. The principal criteria offered relate to three states: futility of continued therapy, depressed neurological status of the patient, and compounding harms from continued clinical care.

The existence of these conditions is certified by clinicians, and thus the permissibility hinges on physician assessment and expertise. Indeed, many of the *fatwās* explicitly mention that the physician is the one who determines whether or not the patient's circumstances meet the criteria for foregoing life-sustaining care. In other words, the justifying conditions for forgoing clinical treatment are reliant upon a physician's assessment of patient's outcome (imminent demise futility, for example) or predicted quality of life (qualitative futility). The *fatwā*s’ dependence on a physician's understanding and expertise may be problematic because clinicians can make errors in prognostication of outcomes and in judging quality-of-life. In some clinical situations, physicians fare no better than chance in predicting a patient's outcome (Geurts et al., 2017). We speculate that jurists may be unaware that medical prognostication near the end-of-life is a tricky science, however the impact of medical uncertainty upon the juridical assessment needs to be studied. In other areas, for example in determining the moral status of seeking medical care, jurists comment that since medical treatment is not certainly curative, one cannot morally obligate patients to seek it (Padela & Qureshi, 2016). In this case it would seem that uncertainty about the restorative potential of clinical treatment would further bolster an argument that forgoing care is permissible.

We found that *fatwās* avoid giving specific clinical examples that meet the threshold of futility. This is understandable given that the jurists appear to defer to clinicians for their judgement on the harms and benefits of continued life-sustaining interventions. At the same time, the ambiguity raises questions about how “futility” ought to be understood and applied within an Islamic ethico-legal framework. Does reliance on a medical understanding limit the term to physiological futility? How does the latter then relate to medical understandings of quality of life? Although the analysis of *fatwās* in relation to pain relief offer some insights into juristic opposition to quality-of-life being an indication for withholding and/or withdrawal of treatment, there is an overwhelming emphasis, within the opinions analysed here, on the role of medical ends in determining futility and little clarity on whether these are the same as religious ends.

When evaluating the cessation of “futile” treatments, however, some scholars do offer a distinction between two categories of end-of-life interventions: life support and ancillary measures. The former refers to treatments such as mechanical ventilation. The latter includes nutrition, hydration, pain control, and antibiotics. The *fatwā*s that addressed this distinction rule that while life support treatment is permissible to stop, ancillary treatment should continue. The review shows that there is less clarity about the indications for the continuation of ancillary treatment. For example, if multiple medical professionals deemed the initiation and/or continuation of ancillary treatment futile, then how should such withholding/withdrawal of care be evaluated within the Islamic ethico-legal paradigm? The Saudi Permanent Committee, when asked regarding the withholding of antibiotic therapy, ruled that it is permissible to initiate such treatment in the hopes that the patient might surpass expectations through divine intervention. The analysis thus indicates that more research needs to be undertaken to evaluate theological reasoning in relation to miracles around end-of-life care and how such reasoning may impact decision-making around the withholding and withdrawal of treatment. Do theological understandings around miracles, identified within the *fatwās,* indicate a type of religious end/goal/commitment relevant to discussions around futility? The analysis here suggests that more research is required to normatively assess the role of medical futility in the Islamic ethico-legal discourse as an indication for the withholding and/or withdrawal of treatment and more specifically whether there are religious ends that ought to be considered when defining and applying notions of futility.

In terms of depressed neurological status, many *fatwās* ruled brain death as an indication for the permissibility of discontinuing life support. Considerations relating to depressed neurological status, in contrast to the notion of “futility,” render an opinion based not on the efficacy of interventions but rather on the physiological state of the patient. In both cases, however, the reliance is on medical knowledge and physician expertise. Again, there is little offered within the *fatwās*, regarding the evaluation of depressed neurological status being reliant on religious ends, for example, the inability of the individual to affect their afterlife (Padela & Mohiuddin, 2015).

In relation to harm, the data indicates that some *fatwās* identified pain that is exacerbated by ongoing treatment, during the final stage of an illness, as an acceptable indication for withholding and/or withdrawing treatment.^36^ Harm here is distinct to futility as the latter relates to limited benefits from medical intervention and the former relates to its injurious aspect. Again, what is meant by harm is poorly understood from these *fatwās.* Is acceptable harm here assessed in biomedical terms where a physical and/or mental evaluation is necessary to justify the withholding/withdrawal of treatment? Or are there religious/spiritual considerations that are implicit in the *fatwās?* For example, would concern for a believer experiencing intractable pain renouncing their faith be an indication for the withholding/withdrawal of treatment? How far if at all do such *fatwās* involve consideration of Islamic theological concepts of human dignity (*karāmah*) and inviolability (*hurmah*) (Padela & Qureshi, 2016) as additional parameters of evaluating harm within the Islamic ethico-legal framework and as a justification or the withholding and withdrawal of treatment? An additional finding is that “harm” within such *fatwās* is distinct to quality-of-life considerations. Pain is offered as a metric of harm within the final stage of an illness, whereas in other clinical contexts it is considered a metric of quality of life and cannot be a justification for withholding/withdrawal of treatment. What constitutes “the final stage of illness,” however, is not clearly characterised and may simply be synonymous with medical evaluation.

This study indicates that more research is also needed to clarify not only the definitions of terms employed by jurists when offering an opinion on bioethical matters, but also the perspectives from which these terms are deployed*.* For example, how should terms like *futility* and *harm* be understood and applied with respect to the perspective of the patient, family, medical professionals and the health care system? Do the *fatwās* analysed here imply universality or do each of these contexts/perspectives require a unique evaluation of such terms? The consideration of scarce resources as an indication to discontinue care, from IFA-I, does highlight at the individual and familial level the acceptability of such a factor for the withholding/withholding of treatment. Here the potential financial harm the patient/family may accrue from intractable debt may be the justification for discontinuing care. However, systemically, what would the application and implications of such *fatwās* be? Can such an ethico-legal permission at the individual level be applied at the population level to absolve the need for providing care for those who cannot afford it? Or would systemic resource considerations require a different evaluation and would the underlying normative values be different to those employed for the individual case e.g. an Islamic ethico-legal notion of justice applied at the state level as it pertains to the provision of adequate and effective end of life care?

While our narrative *fatwā* review provides some clarity regarding the Islamic bioethical stances regarding withdrawal and withholding of life support, our results must be borne in mind in light of several limitations. For one our literature search strategy was predominantly limited to English-language resources easily accessible online. It is possible that *fatwās* in other languages, e.g. Arabic or Urdu, might have provided different views on the moral status of forgoing clinical interventions, or provided different sets of conditions for permissibility. In addition, *fatwās* can be produced in newspaper outlets, in book collections published by local presses, and sometimes only communicated verbally (Arda & Rispler-Chaim, 2010). As such it is simply not possible to comprehensively canvass all verdicts, and our study should be considered a launching point for future research and ethico-legal deliberations. Indeed all *fatwā*-research is subject to a form of publication bias. Aside from limitations due to method, one must also recognise the limitations of *fatwās* as an ethio-legal source. *Fatwās* are generally contingent instruments of Islamic law issued by scholars who are attempting to address novel circumstances and pressing issues that pose a “religious threat” to the questioner. Thus the contextual issues might be prioritised and non-normative rulings issued in order to resolve crises of faith. Consequently, the researcher must be careful not to mistake exceptions to be norms, and the seeker of guidance runs the risk of misapplying contingent *fatwās* out of context. Accordingly our findings must be treated with caution, and, while they accurately represent the literature, not to be considered as offering a declarative Islamic bioethical normative judgement.

## Notes

1 We adopt Prof. Mohamed Fadel's usage of the English term moral theology to refer to the Islamic science of *uṣūl al-fiqh*. See Fadel (2008).

2 For a background on Shari'ah, Islamic Law, and Usul al-Fiqh, see the following overview treatises: Calder, Norman, Joseph A. Kéchichian, Farhat J. Ziadeh, Abdulaziz Sachedina, Jocelyn Hendrickson, Ann Elizabeth Mayer and Intisar A. Rabb. “Law.” In The Oxford Encyclopedia of the Islamic World. Oxford Islamic Studies Online, http://www.oxfordislamicstudies.com.proxy.lib.umich.edu/article/opr/t236/e0473; Vikør, Knut S. “Sharī^c^ah.” In The [Oxford] Encyclopedia of Islam and Law. Oxford Islamic Studies Online, http://www.oxfordislamicstudies.com.proxy.lib.umich.edu/article/opr/t349/e0117; Ziadeh, Farhat J. “Uṣūl al-fiqh.” In The Oxford Encyclopedia of the Islamic World. Oxford Islamic Studies Online, http://www.oxfordislamicstudies.com.proxy.lib.umich.edu/article/opr/t236/e0831. For a more detailed introduction, see the following: Kamali (2011); Hallaq (2016).

3 For a background on fatwas, see Masud, Muhammad Khalid, Joseph A. Kéchichian, Brinkley Messick, Joseph A. Kéchichian, Ahmad S. Dallal and Jocelyn Hendrickson. “Fatwā.” In The Oxford Encyclopedia of the Islamic World. Oxford Islamic Studies Online, http://www.oxfordislamicstudies.com.proxy.lib.umich.edu/article/opr/t236/e0243. To gain insight into the way fatwas are used in the modern context, see the following: Chawki (2010); Van den Branden and Broeckaert (2011, pp. 167–175).

4 “For an introduction to the field of Islamic bioethics,” see the following: Padela (2007, pp. 169–178); Padela (2013, pp. 65–80); Gatrad and Sheikh (2001, pp. 72–75).

5 “About Mufti Ebrahim Desai,” Dural Iftaa, https://daruliftaa.net/about-mufti-ebrahim-desai/.

6 “Islamic Code of Medical Ethics,” accessed 1 October 2016, http://islamset.net/ethics/code/index.html.

7 “Islamic Fiqh Council.” Muslim World League, 2019, accessed August 26, 2019, https://themwl.org/en/المجمع-الفقهي-الإسلامي-0.

8 “A Brief Report,” 2014, accessed 1 October, 2016, http://www.ifa-india.org/english.php?do=home&pageid=IFA_introduction.

9 “The Permanent Committee for Islaamic Research & Verdicts,” 1999, http://www.fatwa-online.com/permanent-committee/.

10 “About Us: Madrasah Inaa'miyyah,” http://www.alinaam.co.za/about-us/.

11 “European Council for Fatwa and Research,” The Oxford Encyclopedia of the Islamic World, Oxford Islamic Studies Online, http://www.oxfordislamicstudies.com/article/opr/t236/e0935?_hi=2&_pos=5.

12 “About Us: Darul Uloom Zakariyya,” Darul Uloom Zakariyya, http://duz.co.za/index.php/home.

13 “About Us: Jamiatul Ulama,” Jamiatul Ulama, https://jamiat.org.za/about-us/.

14 Further details e.g. date of birth about Hani al-Jubayr were unavailble to researchers at the time of this study

15 “Sheikh Dr Yusuf Al Qaradawi,” theMuslim500, https://www.themuslim500.com/profiles/sheikh-dr-yusuf-al-qaradawi/.

16 “Ali Gomaa,” Berkley Center for Religion, Peace, and World Affairs, n.d., https://berkleycenter.georgetown.edu/people/ali-gomaa.

17 “Islam Question and Answer,” Islamqa, https://islamqa.info.

18 “About Mufti Ebrahim Desai.”

19 “About Us: Dural Iftaa,” Dural Iftaa, http://www.daruliftaa.com/aboutus.

20 “Fatwa Center of America,” Ummah Dictionary, http://ummahdirectory.co.uk/listings/fatwa-center-of-america/.

21 “Question #33545,” Manners: Mercy Killing, IslamiCity, 2009.

22 “Ali Gomaa – Grand Mufti of Egypt,” (2011/12/20/23:19:27 2011), http://www.aligomaa.net/fatwacollection.html.

23 “Fatwa No. 17461,” AskImam, 2009, http://askimam.org/public/question_detail/17461.

24 “Question #33545.”

25 “Fatwa no. 12086,” The Permanent Committee for Scholarly Research and Ifta, 1989, http://alifta.com/Fatawa/FatawaSubjects.aspx?languagename=en&View=Page&HajjEntryID=0&HajjEntryName=&RamadanEntryID=0&RamadanEntryName=&NodeID=4693&PageID=9767&SectionID=7&SubjectPageTitlesID=10021&MarkIndex=1&0#Tousetheresuscitator.

26 “Manners: Mercy Killing,” IslamiCity, http://www.islamicity.org/qa/action.lasso.asp?-db=services&-lay=Ask&-op=eq&number=33545&-format=detailpop.shtml&-find.

27 Muhammad ibn Adam, “Stopping Medical Treatment when there is no Expected Benefit | daruliftaa.com,” http://www.daruliftaa.com/node/5899?txt_QuestionID=.

28 “Fatwa no. 18347,” Ask Imam, 2009, http://askimam.org/public/question_detail/18347.

29 “Question #33545.”

30 “Yusuf al-Qaradawi,” Islamopedia, updated 2011/12/20/23:59:02, 2011, http://www.islamopediaonline.org/fatwa/what-islamic-stance-euthanasia.

31 “Euthanasia: Types and Rulings,” European Council for Fatwa and Research, 2003, https://archive.islamonline.net/?p=1410.

32 “Fatwa No. 17461.”

33 “Yusuf al-Qaradawi.”

34 ibn Adam, “Stopping Medical Treatment when there is no Expected Benefit | daruliftaa.com.”

35 “Yusuf al-Qaradawi.”

36 “Yusuf al-Qaradawi.”
